# Ultrasensitive detection of 5-hydroxymethylcytosine in genomic DNA using a graphene-based sensor modified with biotin and gold nanoparticles

**DOI:** 10.1016/j.mtbio.2024.101123

**Published:** 2024-06-11

**Authors:** Habibulla Imran, Hyun-ji Lee, Asrar Alam, Jungeun An, Myunggon Ko, Sooman Lim

**Affiliations:** a Department of Flexible and Printable Electronics, LANL-JBNU Engineering Institute-Korea, Jeonbuk National University, Jeonju 54896, Republic of Korea; b Department of Biological Sciences, Ulsan National Institute of Science and Technology, Ulsan, 44919, Republic of Korea; c Mycronic AB, Nytorpsvägen 9, Täby, 183 53 Sweden; d Wallenberg Initiative Materials Science for Sustainability (WISE), Department of Fibre and Polymer Technology, School of Engineering Sciences in Chemistry, KTH Royal Institute of Technology, Teknikringen 56, Stockholm, 10044, Sweden; e Department of Life Sciences, Jeonbuk National University, 567 Baekje-daero, Jeonju, 54896, Republic of Korea

**Keywords:** 5hmC, Cancer biomarker, Electrochemical quantification, Graphene-based sensor, Gold nanoparticle, Graphene functionalization

## Abstract

Ten-eleven translocation (TET) proteins orchestrate deoxyribonucleic acid (DNA) methylation-demethylation dynamics by oxidizing 5-methylcytosine to 5-hydroxymethylcytosine (5hmC) and are frequently inactivated in various cancers. Due to the significance of 5hmC as an epigenetic biomarker for cancer diagnosis, pathogenesis, and treatment, its rapid and precise quantification is essential. Here, we report a highly sensitive electrochemical method for quantifying genomic 5hmC using graphene sheets that were electrochemically exfoliated and functionalized with biotin and gold nanoparticles (Bt-AuNPs) through a single-step electrical method. The attachment of Bt-AuNPs to graphene enhances the specificity of 5hmC-containing DNA and augments the oxidation of 5hmC to 5-formylcytosine in DNA. When coupled to a gold electrode, the Bt-AuNP-graphene-based sensor exhibits exceptional sensitivity and specificity for detecting 5hmC, with a detection limit of 63.2 fM. Furthermore, our sensor exhibits a remarkable capacity to measure 5hmC levels across a range of biological samples, including preclinical mouse tissues with varying 5hmC levels due to either TET gene disruption or oncogenic transformation, as well as human prostate cancer cell lines. Therefore, our sensing strategy has substantial potential for cancer diagnostics and prognosis.

## Highlights

• One-step electrochemical process for simultaneous exfoliation and functionalization of graphene with biotin and gold nanoparticles.
• A highly sensitive, graphene-based sensor for label-free detection of 5hmC in DNA with a remarkably low detection limit of 63.2 fM.
• Robust 5hmC detection across a variety of biological samples, including preclinical murine tissues and human prostate cancer cell lines.
• Consistent 5hmC quantification results compared to those obtained by other assays.

## Graphical abstract

**Figure:**
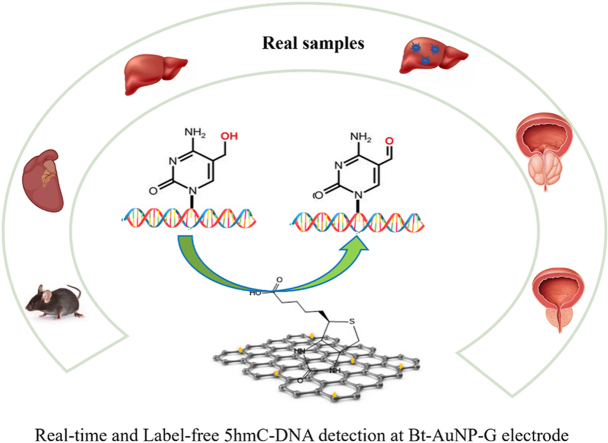

## 1 Introduction

Aberrations in epigenetic mechanisms play crucial roles in cancer development by affecting gene expression and critical physiological processes [[1], [2], [3]]. Ten-eleven translocation (TET) proteins, including TET1, TET2, and TET3 regulate deoxyribonucleic acid (DNA) methylation by converting 5-methylcytosine (5 mC) to 5-hydroxymethylcytosine (5hmC) [4]. The dysregulation of TET proteins leading to a reduction in 5hmC is commonly observed in various cancers, including hematological neoplasms [5,6] such as acute myeloid leukemia, myelodysplastic syndromes, B-cell or T-cell lymphomas, and solid tumors in different tissues such as the skin, brain, liver, lung, breast, pancreas, colon, and prostate [[7], [8], [9], [10], [11], [12], [13]]. Impaired TET function significantly facilitates cancer initiation and progression, highlighting TET proteins as essential tumor suppressors [5,14]. Reduced 5hmC levels correlate with poor prognosis and tumor aggressiveness [[15], [16], [17]]. Moreover, distinct 5hmC profiles present a promising avenue for non-invasive cancer diagnostics and disease monitoring [18], and changes in the 5hmC levels function as predictive biomarkers for disease trajectory and therapeutic outcomes [16,17,[19], [20], [21], [22]]. The upregulation of TET enzymatic activity in immunotherapy has yielded remarkable therapeutic efficacy [23,24], underscoring the potential of 5hmC quantification in identifying candidates suitable for such treatment. Collectively, these results underscore the value of 5hmC as a biomarker for cancer detection, prognosis, and personalized treatment strategies.

Several detection methods have been employed to identify 5hmC within the genomic landscape. These range from traditional chromatography [[25], [26], [27]], enzyme-based techniques utilizing endonucleases [28] or radiolabeled glucose [29], and antibody-based methods [[30], [31], [32]] to advanced techniques such as bisulfite conversion coupled with next-generation sequencing [33]. However, the translation from research to clinical application is hindered by significant challenges; existing methods are often time-consuming, labor-intensive, expensive, and fraught with potential errors due to incomplete chemical conversion and enzymatic biases. Nanomaterial-based biosensors, which utilize principles such as surface plasmon resonance and fluorescence resonance energy transfer [[34], [35], [36], [37]], have improved the efficiency and cost-effectiveness of 5hmC detection. However, these methods fail to achieve the stringent selectivity and sensitivity required for clinical diagnostics. It is crucial to develop advanced detection methods that not only meet clinical standards but also exhibit superior selectivity, reproducibility, and accuracy, particularly for pre-clinical and clinical samples. Addressing these requirements will ensure that 5hmC detection can accurately inform clinical decisions, leading to improved patient outcomes.

Graphene is a carbon material with a *sp*^*2*^-hybridized two-dimensional (2D) structure and its exceptional properties, including superior electrical, thermal, and mechanical characteristics, have made it a transformative material in healthcare and biosensing [[38], [39], [40], [41], [42]]. Despite its potential, cost-effective production of high-quality graphene remains a significant challenge [43]. Among the various synthesis methods, such as chemical synthesis, chemical exfoliation, mechanical exfoliation, chemical vapor deposition (CVD), microwave synthesis, nanotube unzipping, and atomic force microscope cantilevering [38,[44], [45], [46], [47], [48], [49]], the electrochemical exfoliation of graphite has emerged as a highly effective method due to its simplicity, high efficiency, and eco-friendliness [43]. Ionic liquid-based exfoliation often results in low quantities of graphene with small lateral dimensions (<5 μm) and compromised electronic integrity [[50], [51], [52]], whereas exfoliation in acidic electrolytes can produce higher quality graphene sheets, although it may impair electrical properties due to excessive oxidation [[53], [54], [55]]. Moreover, the functionalization of graphene to modify its surface properties, electrical conductivity, and band gap has been identified as a means of enhancing its biosensing capabilities [56]. For example, graphene can be functionalized with gold nanoparticles (AuNPs) via galvanic exchange, thiol chemistry, and citrate reduction [[57], [58], [59]]. Electrochemically exfoliated graphene surfaces can be decorated with AuNPs via electrochemical deposition [59]. However, traditional functionalization techniques are generally laborious and time-intensive [[60], [61], [62], [63], [64], [65]].

Herein, we introduce a one-step synthesis of biotin (Bt)-AuNP-functionalized graphene (Bt-AuNP-G) using pencil graphite in an aqueous medium. We subjected the synthesized Bt-AuNP-G to rigorous physical and electrochemical characterizations, confirming its ultrahigh sensitivity and specificity for genomic 5hmC detection. Furthermore, when coupled to a gold electrode (AuE), Bt-AuNP-G demonstrated consistent and robust detection of 5hmC in a variety of authentic biological samples from primary mouse tissues, with and without TET enzymes, hepatocellular carcinoma specimens, and human prostate cancer cell lines. Our approach not only simplifies the production of functionalized graphene but also has significant potential for clinical applications, particularly in early cancer detection and prognosis evaluation.

## 2 Materials and methods

Details regarding the materials (2.1), one-pot direct electrochemical exfoliation and biotin-AuNP functionalization of graphene using a pencil graphite rod (2.2), sensor device fabrication (2.3), characterization of sensor materials (2.4), electrochemical measurements (2.5), synthesis of oligonucleotides containing distinct cytosine variants (2.6), conditional deletion of *Tet* genes in mouse hepatocytes (2.7), generation of mouse models with primary hepatocellular carcinoma (2.8), extraction of genomic DNAs and quantification of 5hmC levels (2.9), cell culture (2.10) and statistical analyses (2.11) are available in the Supplementary Information.

## 3 Results and discussion

### 3.1 Concurrent electrochemical exfoliation and functionalization of graphene layers

**Scheme 1:**
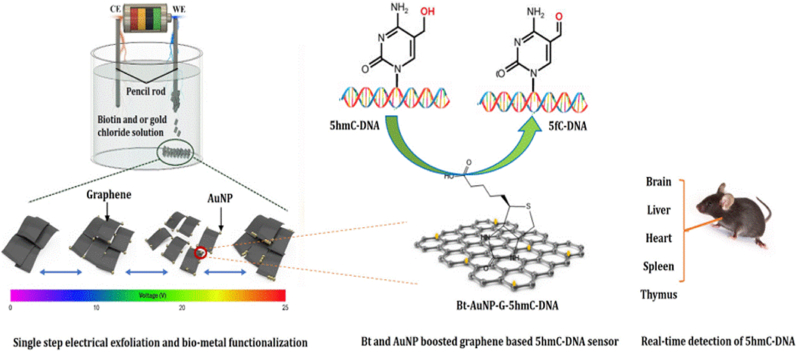
Single-step electrochemical exfoliation and functionalization of graphene for highly sensitive detection of 5hmC in DNA.

The fabrication process begins with the anodic electrochemical exfoliation of graphene sheets from a graphite rod immersed in a solution containing Bt and gold chloride (Scheme 1). When voltages are applied to initiate the electrochemical reactions, delamination and isolation of graphene layers from graphite occur, which are subsequently integrated with Bt-AuNPs to yield functionalized graphene (termed Bt-AuNP-G) (Figs. S1 and S2). To determine the optimal voltage for this process, the influence of voltages ranging from 10 to 25 V on the resulting graphene structure was assessed. The structures of pencil graphite and Bt-AuNP-G synthesized under different conditions were assessed using field emission scanning electron microscopy (Fig. S2). While the initial pencil graphite exhibited a thick flake-like structure, the exfoliated and functionalized graphene exhibited thin and smooth sheet-like structures at all the voltages tested. During exfoliation, the Bt-AuNPs may intercalate into the anodic electrode, causing the layers to separate into individual graphene sheets. In this process, the extent of exfoliation and graphene thickness appeared to vary depending on the voltage. At 15 V, delamination occurred, but heterogeneous graphene layers with variable thicknesses were generated. However, at 20 V, a more homogeneous graphene layer was produced, with Bt-AuNPs homogeneously deposited on the surface of the graphene sheets. Bt-AuNPs may increase the number of active sites and sensitivity. Increasing the voltage further to 25 V resulted in the aggregation of the graphene surface, forming a thin, interconnected graphene layer. The resulting exfoliated graphene solution remained stable for up to 7 days (Fig. S3).

Cyclic voltammetry (CV) was subsequently used to evaluate the electrochemical characteristics of AuEs modified with either pencil graphite or Bt-AuNP-G produced under different voltages (Fig. S4). The measurements were conducted in a redox solution of a 10 mM K_3_[Fe(CN)_6_]/K_4_[Fe(CN)_6_] in 100 mM KCl, maintaining a scan rate of 50 mVs^−1^. Relative to pencil graphite, all Bt-AuNP-G variants exhibited increased peak currents with a simultaneous decrease in peak potential separation (ΔE_p_), as detailed in Table S1. This enhanced electrical performance of Bt-AuNP-G may be attributed to its conductive pi-electron framework and striated architecture, which augment the peak currents on AuEs. Remarkably, the 20 V exfoliation condition yielded the highest anodic oxidation peak current and the lowest ΔE_p_ values, indicative of rapid exfoliation, enhanced conductive properties, and a reduced number of graphene layers (Table S1). These findings collectively suggest that exfoliation at 20 V results in optimal morphological features, prompting the use of this condition for subsequent studies.

### 3.2 Physical characterization of the functionalized graphene

**Fig. 1:**
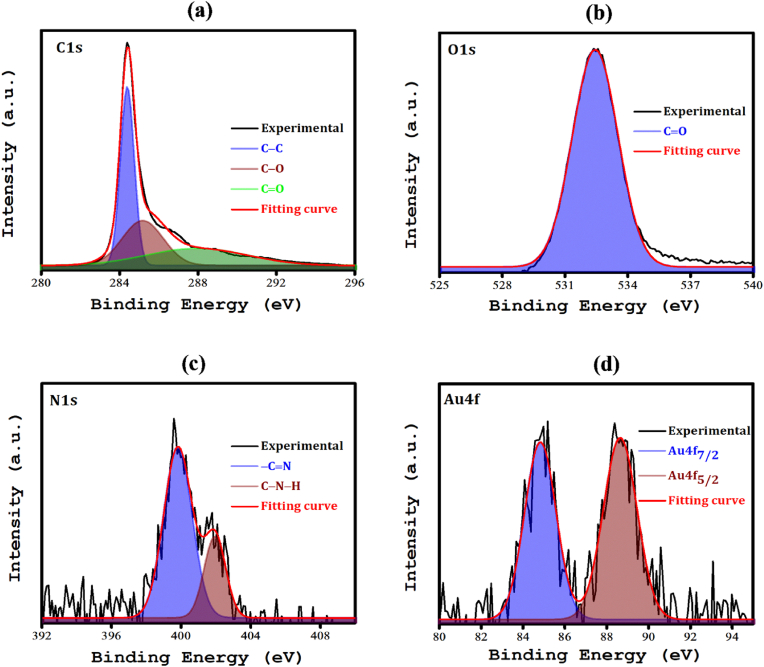
XPS spectrum of electrochemically exfoliated graphene functionalized with biotin and gold nanoparticles (Bt-AuNP-G). (For interpretation of the references to colour in this figure legend, the reader is referred to the Web version of this article.)

The physical properties of graphene functionalized with Bt and/or AuNPs (Bt-G, AuNP-G, and Bt-AuNP-G) were assessed via X-ray photoelectron spectroscopy (XPS), to analyze their chemical compositions and functional groups. Fig. 1 and S5show the core-level XPS spectra of C1s, O1s, N1s, and Au4f. The C1s spectra of all samples showed three distinct peaks at 284.3, 286.2, and 288.6 eV corresponding to *sp*^*2*^-hybridized carbon (C–C), epoxide (C–O), and carbonyl (C=O) moieties of graphene, respectively (Figs. S5a and d and Fig. 1a) [66]. Notably, Bt-AuNP-G exhibited elevated peak intensities and binding energies, with a significant atomic percentage of 85.66 % for C1s, exceeding those of Bt-G at 76.51 % and AuNP-G at 71.95 %. This might be due to the exposure of highly reactive carbonyl groups and Au species during the functionalization process in the solution of Bt and AuCl_3_, leading to a reduction in the epoxide and carbonyl constituents on the surface.

In the O1s spectra, there were two principal peaks at 530.3 and 532.2 eV (Figs. S5b and e and Fig. 1b), which were attributed to C=O and C–O, respectively [67]. For Bt-AuNP-G, there was a marked decrease in the 530.3 eV peak, indicating the selective abatement of C=O functionalities upon integration with Bt and AuNPs. Furthermore, there was a decrease in the 532.2 eV peak, indicating the loss of oxygen and possibly carbon. The N1s spectra showed peaks at 399.6 and 401.7 eV (Figures S5c and Fig. 1c), associated with nitrogen situated between aromatic rings (-C=N) and linked to amino groups (C–N–H)), respectively [[68], [69], [70]], with Bt-G and Bt-AuNP-G exhibiting 1.23 % and 0.83 % of N1s, respectively. The 399.6 eV peak in both the Bt-G and Bt-AuNP-G samples can be ascribed to functionalization with Bt, which comprises NH bonds. The Au4f region in AuNP-G and Bt-AuNP-G samples exhibited peaks at 83.8 eV (Au4f_7/2_) and 89.9 eV (Au4f_5/2_) (Figures S5f and Fig. 1d), confirming the crystalline gold structure [71]. These findings indicate that the AuNPs crystallized and were fixed to the graphene surface. Moreover, Bt-AuNP-G exhibited a reduced peak intensity and a binding energy shift, along with an increased atomic percentage of carbon and decreased oxygen, nitrogen, and gold contents. These functional groups typically congregate at graphene edges, leading to in-plane defects and disorders [72].

**Fig. 2:**
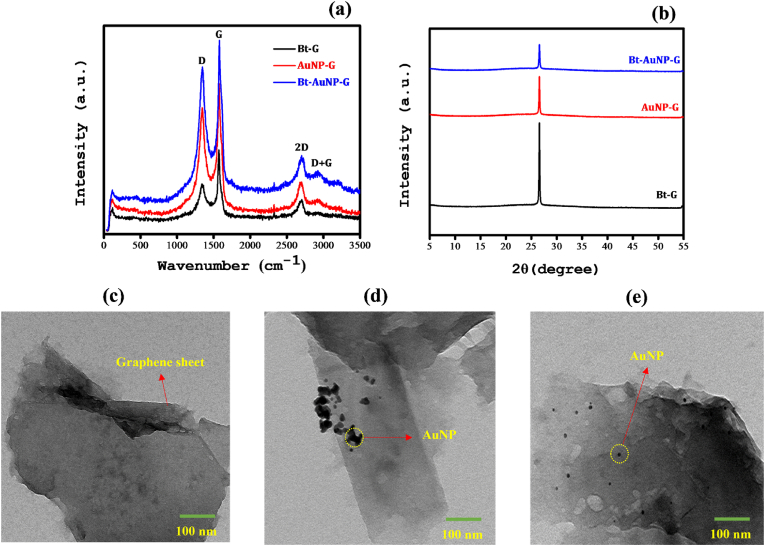
(a) Micro-Raman spectrum, (b) XRD pattern, and (c–e) TEM images of Bt-G, AuNP-G, and Bt-AuNP-G.

Raman spectroscopy analysis revealed distinct peaks at 1356, 1576, and 2687 cm^−1^, corresponding to the D, G, and 2D bands, respectively (Fig. 2a). The D band signifies a defect-induced resonance, the G band is indicative of the E2g phonon of *sp*^*2*^ carbon atoms, and the 2D band represents a second-order process involving D-band transitions [73,74]. The calculated *I*_*D*_*/I*_*G*_ ratios of Bt-G, AuNP-G, and Bt-AuNP-G were 0.53, 0.79, and 0.85, respectively. Bt-AuNP-G exhibited the highest *I*_*D*_*/I*_*G*_ ratio, indicating the presence of more surface defects. Using the equation *L*_a_ = 2.4 × 10^−10^ λ^4^ [*I*_D_/*I*_G_]^−1^, we determined the crystallite sizes to be approximately 31, 21, and 19 nm for Bt-G, AuNP-G, and Bt-AuNP-G-, respectively, indicating that Bt-AuNP-G had the smallest crystallite sizes, which is consistent with its higher degree of surface imperfections [59], compared to Bt-G and AuNP-G.

X-ray diffraction analysis revealed that Bt-G, AuNP-G, and Bt-AuNP-G each displayed a pronounced diffraction peak at 2θ = 26.5°, indicative of the crystalline graphite (002) plane (Fig. 2b). No additional diffraction peaks corresponding to Au or N were observed. The XPS results revealed an exceptionally low atomic percentage of Au and N on the graphene surface (Fig. 1 and S5), which may account for the absence of additional diffraction peaks in the composites. Morphological analysis of the functionalized graphene variants using transmission electron microscopy confirmed the uniform 2D sheet-like structure across all the graphene modifications (Fig. 2c–e). Bulky AuNPs anchored at the periphery of the graphene sheets were observed for AuNP-G. In contrast, the Bt-AuNP-G samples exhibited a uniform 2D structure with smaller AuNPs evenly dispersed across the graphene surface, potentially enhancing the active surface area and electrical conductivity.

### 3.3 Electrochemical detection of 5hmC in DNA on Bt-AuNP-G modified surface

Given the critical role of 5hmC as a cancer biomarker, we investigated the potential of our electrochemically exfoliated and functionalized graphene for detecting 5hmC in DNA [[15], [16], [17], [18], [19], [20], [21], [22]]. Oligonucleotides harboring 5hmC (5hmC-DNA) were synthesized as described in Section 2.6and immobilized onto AuEs pre-modified with bare graphene (G, Fig. S6a), biotinylated graphene (Bt-G,) Fig. S6b, AuNP-G (Fig. S6c), and Bt-AuNP-co-functionalized graphene (Bt-AuNP-G, Fig. S6d) via drop-casting. Following this, CV was performed in 100 mM KCl with the K_3_[Fe(CN)_6_]/K_4_[Fe(CN)_6_] redox probe, a method known for its sensitivity in detecting biomolecular interactions, particularly with DNA [75]. As shown in Figs. S6a–d, 5hmC-DNA exhibits a notably stronger affinity for the AuE-Bt-AuNP-G surface than for the other surfaces. This suggests that the dual functionalization of graphene with both AuNPs and Bt may significantly increase the density and specificity of the DNA-binding sites. Conversely, the AuE-G surface showed weaker DNA interactions, likely due to its inert nature and lack of specific binding sites. Overall, these findings confirmed the interactive potential of 5hmC-DNA with the AuE-Bt-AuNP-G surface. Notably, DNA containing unmodified cytosine (C-DNA) also displayed adsorption levels on the AuE-Bt-AuNP-G surface comparable to those observed for 5hmC-DNA (Fig. S6e), suggesting that CV analysis using the K_3_[Fe(CN)_6_]/K_4_[Fe(CN)_6_] redox probe is not suitable for the selective detection of 5hmC-DNA.

**Fig. 3:**
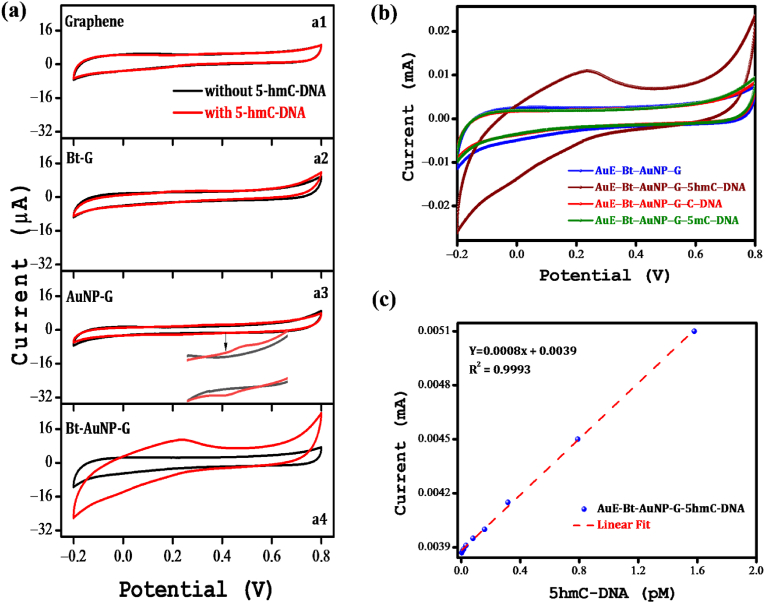
(a) CV analysis of AuE modified with (a1) graphene, (a2) Bt-G, (a3) AuNP-G, and (a4) Bt-AuNP-G with (*red*) or without (*black*) 5hmC-DNA. (b) Sensor selectivity demonstrated by recording CV using Bt-AuNP-G-modified AuE after immobilization of DNA containing C (*red*), 5 mC (*green*), or 5hmC (*brown*). (c) Graph showing the impact of 5hmC levels on the LSV oxidation peak current according to the concentration of 5hmC-DNA. The orange dashed line represents the linear regression curve. (For interpretation of the references to colour in this figure legend, the reader is referred to the Web version of this article.)

An electrochemical oxidation approach was utilized to distinguish between 5hmC-DNA and C-DNA [76]. Fig. 3a shows CV measurements that were performed in a 100 mM KCl solution, spanning a potential range from −0.2 V to 0.8 V at a scan rate of 50 mVs^−1^ using the electrodes described above, both in the presence and absence of immobilized 5hmC-DNA. Despite the inherent capacity of graphene for DNA adsorption, the electrodes modified with graphene (**a1**) exhibited no observable redox reactions, implying that graphene alone is insufficient to facilitate the oxidation process. Upon functionalization of graphene with Bt (**a2**), a minor reduction in non-Faradaic peak current within the −0.2 V–0.2 V range was observed, reflecting the DNA adsorption capability of Bt; however, no redox activity was detected. Conversely, the addition of AuNPs to graphene (**a3**) resulted in a modest yet noticeable redox peak attributable to the electrocatalytic properties of AuNPs. This suggests that AuNPs may act as electrocatalysts, enhancing electron transfer processes and facilitating oxidation of the hydroxyl group in 5hmC to form 5-formylycytosine (5 fC), which simultaneously occurs with the release of two protons per 5hmC. Subsequently, 5 fC was reduced to 5hmC during the reverse scan, producing an intense reverse peak. Intriguingly, the Bt-AuNP-G-modified AuE exhibited a substantially higher oxidation peak current (0.0112 mA) and more reduced oxidation potential (230 mV) than those modified with AuNP-G (0.0029 mA, 360 mV) alone (Fig. 3a, a4). This implies that this dual modification acts as an effective catalyst, allowing for the adsorption and oxidation of more 5hmC-DNA molecules on the electrode surface.

To assess the selectivity of the fabricated sensor, the Au-Bt-AuNP-G electrode was exposed to oligonucleotides containing cytosine (C-DNA) or 5-methylcytosine (5 mC-DNA). Interestingly, they did not exhibit the same oxidation characteristics as those of 5hmC-DNA (Fig. 3b). Because the nucleotide sequences of all oligonucleotides used were identical, except for C, 5 mC, and 5hmC as sources of cytosine variants, these results underscore the specificity of the Bt-AuNP-G-modified electrodes in facilitating the selective oxidation of 5hmC. These findings demonstrate that our Bt-AuNP-G-modified AuE demonstrates an enhanced ability to specifically detect 5hmC-DNA, eliminating the need for elaborate pretreatment or labeling [77,78]. Consequently, we selected Bt-AuNP-G-modified AuEs for further optimization studies.

### 3.4 Reproducibility, stability, and limit of detection of the sensor device

To evaluate the reproducibility of 5hmC detection, we prepared four separate electrodes immobilized with Bt-AuNP-G-5hmC-DNA using identical protocols and analyzed their CV responses. The CV responses of the independently prepared electrodes exhibit high reproducibility (Fig. S7). Additionally, the CV tests conducted daily with an electrode stored at 4 °C in a KCl solution reveal a stable peak current for the initial six days, with a slight reduction starting on day 7 (Fig. S8), indicating that the electrode maintained its stability for at least six days.

Next, we assessed the impact of various concentrations of 5hmC-DNA on the oxidation peak current over 0.005–2.5 ng of 5hmC-DNA (Fig. 3c). Given that the synthesized 5hmC-DNA had a 5hmC ratio of 0.632 pmol/ng (Experimental section 2.6), the assay spanned a 5hmC range of 0.00316–1.58 pmol. The peak current showed a robust linear correlation (correlation coefficient = 0.9993) with the amount of 5hmC-DNA (Fig. 3c). The limit of detection (LOD), determined in accordance with IUPAC recommendations from 1994 [75], was found to be 63.2 fM, and the sensitivity of the sensor was 0.8 μA/pM. This LOD is comparable to or even an order of magnitude lower than those reported in prior studies (Table S2). These results validate the reproducibility, stability, and sensitivity of our sensor device in detecting 5hmC.

### 3.5 Quantification of genomic 5hmC levels in primary murine tissues

**Fig. 4:**
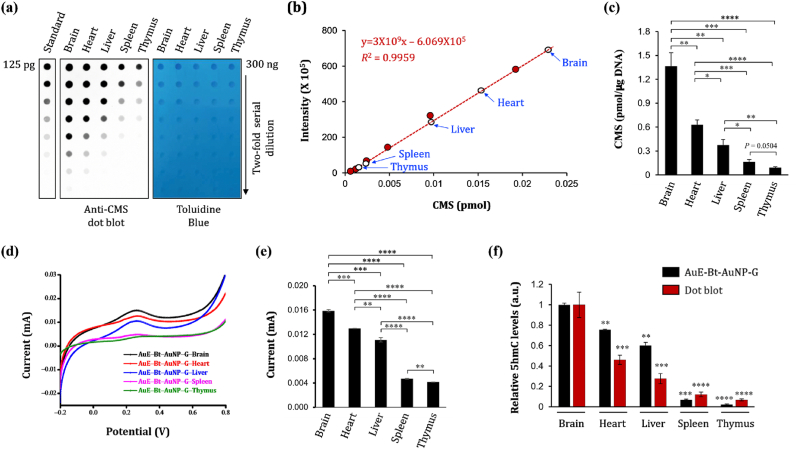
**(**a) Dot blot assay for the indicated tissues from C57BL/6 mice. Toluidine blue staining confirms equal DNA loading. A representative result from three experiments is shown. (b) Quantification of 5hmC levels using standard oligonucleotide with known CMS content. Open circles represent mean signal intensities. (c) Summary of 5hmC levels from the data in (a) and (b). (d) Current response vs. potential graph for genomic DNAs from tissues in (a). A representative result from three experiments, conducted in 100 mM KCl solution at a scan rate of 50 mVs^−1^ scan rate. (e) A summary of peak current values from (d), where each dot represents the mean of three independent experiments. (f) Comparison of results from AuE-Bt-AuNP-G sensor with those obtained via CMS dot blot. 'Relative 5hmC level (a.u.)' indicates the relative amount of 5hmC in each sample, normalized to the level of the first sample in each assay, which was set to 1. Data: Mean ± SEM; *p ≤ 0.05, **p ≤ 0.01, ***p ≤ 0.001, and ****p ≤ 0.0001 (unpaired Student's t-test). (For interpretation of the references to colour in this figure legend, the reader is referred to the Web version of this article.)

We subsequently evaluated the ability of our sensor to detect 5hmC-DNA in actual biological samples, specifically from various primary murine tissues. Genomic DNA was extracted from the brain, heart, liver, spleen, and thymus of 8–12-week-old C57BL/6 mice. These DNA samples were then physically fragmented and treated with bisulfite to convert 5hmC to cytosine-5-methylene-sulfonate (CMS), according to established protocols [30,32,76]. We opted to use anti-CMS antibodies rather than anti-5hmC antibodies because the former are less reliant on the density of 5hmC in the genome, thus enabling accurate quantification using small amounts of DNA [30]. Dot blot assays with anti-CMS antibodies revealed distinct levels of CMS (i.e. 5hmC) across different tissues (Fig. 4a).

For quantification, we performed an additional dot blot with a standard oligonucleotide containing a known concentration of CMS (Fig. 4a, *left panel*) and created a standard curve from the signal intensities relative to the CMS concentration [30,32]. Using this curve, we calculated the 5hmC levels in the tissue DNA samples: brain: 1.365 ± 0.170 pmol/μg, heart: 0.630 ± 0.062 pmol/μg, liver: 0.377 ± 0.068 pmol/μg, spleen: 0.165 ± 0.031 pmol/μg, and thymus: 0.092 ± 0.013 pmol/μg (Fig. 4b and c).

CV analyses of these samples using our sensor produced comparable results, with a higher 5hmC content corresponding to stronger current responses. The brain, heart, liver, spleen, and thymus samples yielded results of (0.016 ± 0.000203) mA, (0.013 ± 0.00047) mA, (0.011 ± 0.000361) mA, (0.005 ± 0.000093) mA, and (0.004 ± 0.000033) mA, respectively (Fig. 4d and e). The current responses are predominantly attributed to the 5hmC content, which differs despite having identical nucleotide sequences and similar 5 mC levels in these DNA samples [79]. The quantification of 5hmC using the standard curve in Fig. 3c also aligned with the CMS dot blot assay results (Fig. 4f), thereby confirming the utility of the sensor for analyzing 5hmC in primary tissues.

### 3.6 5hmC detection in preclinical animal models

We further validated the efficacy of our sensor in quantifying the 5hmC levels in two in vivo preclinical models. To achieve this, we utilized mice with targeted disruptions of all three *Tet* genes in the liver by crossing *Tet* triple-floxed (*Tet1/2/3*^*fl/fl*^) mice with albumin-Cre transgenic mice [78]. Previous research using dot blot analysis demonstrated that Tet triple knockout (TKO; *Tet1/2/3*^*fl/fl*^ *Albumin-Cre*) mice had significantly reduced levels of 5hmC (approximately 3.7-fold) in their livers compared with wild-type (WT *Tet1/2/3*^*fl/fl*^) mice [76].

CV conducted with the AuE-Bt-AuNP-G-based sensor mirrored these findings. The WT DNA samples exhibited consistently higher current responses than TKO samples (0.0149 ± 0.0004 mA for WT vs. 0.0060 ± 0.0001 mA for TKO) (Figs. S9a and b). These genomic DNA samples exhibited identical nucleotide sequences and 5 mC content but different 5hmC levels. Thus, the variation in the current response was primarily attributed to the 5hmC concentration. Moreover, the 5hmC levels calculated from the current responses correlated closely with those from previous reports (Fig. S9c), confirming the precision and reliability of our sensor.

This sensor was also applied to murine models of hepatocellular carcinoma induced by diethylnitrosamine [80], a known carcinogenic agent. Our previous research indicated that tumor (T) tissues exhibited a significant reduction in 5hmC compared to adjacent non-tumor (NT) tissues [76,78]. Consistent with these findings, CV assessments showed that NT samples produced stronger current responses (0.0113 ± 0.0003 mA) than T samples (0.0067 ± 0.0002 mA) (Figs. S9d and e). This observation is consistent with the results of previous studies (Fig. S9f).

### 3.7 5hmC detection in human cancer cell lines

**Fig. 5:**
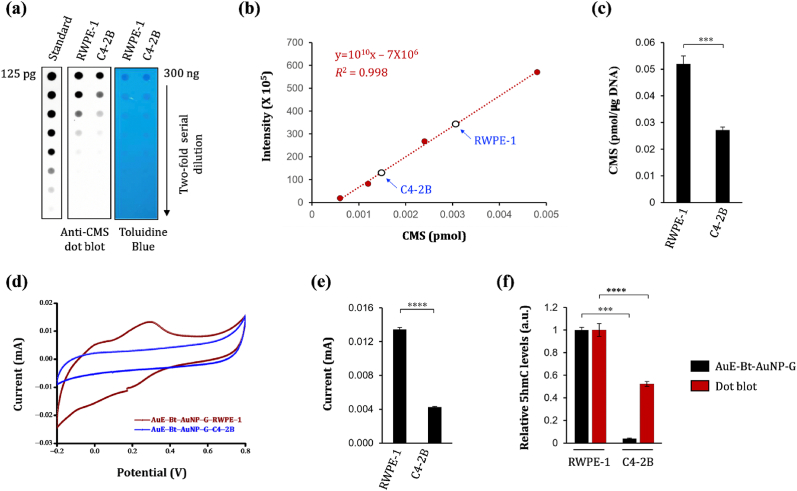
**(**a) Dot blot assay showing the 5hmC levels in RWPE-1 and C4–2B cells. Toluidine blue staining confirms equivalent DNA loading. (b) Quantification of 5hmC using a standard oligonucleotide with a known CMS content. Open circles represent mean signal intensities for the cell lines in (a). (c) Summary of results from (a) and (b). (d) CV analysis on genomic DNAs from the aforementioned cell lines. (e) Summary of peak current values from (d). (f) Comparison of the results using the AuE-Bt-AuNP-G sensor and dot blot. 'Relative 5hmC level (a.u.)' indicates the relative amount of 5hmC in each sample, normalized to the level of the first sample in each assay, which was set to 1. Data: Mean ± SEM; ***p ≤ 0.001 and ****p ≤ 0.0001 (unpaired Student's t-test). (For interpretation of the references to colour in this figure legend, the reader is referred to the Web version of this article.)

To assess the utility of the sensor for distinguishing 5hmC levels in human cancer, genomic DNA was extracted from C4–2B (a prostate cancer cell line) and RWPE-1 (a normal prostate epithelial cell line). Dot blot assays revealed that RWPE-1 cells (0.0520 ± 0.0030) pmol/μg) had higher 5hmC levels than C4–2B cells (0.0272 ± 0.0011) pmol/μg) (Fig. 5a–c). CV analyses using the uE-Bt-AuNP-G sensor corroborated these results, indicating that DNA from RWPE-1 elicited significantly stronger current responses ((0.0134 ± 0.0002) mA) than that from C4–2B ((0.0043 ± 0.000049) mA) (Fig. 5d and e), reflecting higher 5hmC content in RWPE-1 cells. The results obtained from both the AuE-Bt-AuNP-G sensor and CMS dot blot were consistent (Fig. 5f), further confirming that the developed sensor is capable of detecting 5hmC modifications in clinical samples, including cancer specimens. The anti-CMS antibody used in the dot blot recognizes hydroxymethylated DNA with an average sensitivity of one 5hmC per 201 base pairs [30]. Additionally, the 5hmC levels in the dot blot were detected using enhanced chemiluminescence. As a result, the anti-CMS dot blot is less sensitive than our electrochemical method. Therefore, the discrepancies in the fold reduction observed between the electrochemical measurement and the anti-CMS dot blot in Fig. 5f likely arise from the inherent differences in sensitivity and detection principles of the two methods.

## 4 Conclusion

In this study, we report an eco-friendly graphene-based sensor meticulously engineered for the precise detection of 5hmC in genomic DNA. This sensor was produced via the anodic electrochemical exfoliation of graphite rods with simultaneous functionalization with Bt and AuNPs through a single-step electrical method. The incorporation of AuNPs onto graphene sheets played a critical role in enhancing the oxidation of 5hmC to 5 fC, thereby enabling the selective detection of 5hmC-DNA. Assisted by Bt, this integration promoted the specific binding and oxidation of 5hmC-DNA on the electrode, culminating in the formation of a Bt-AuNP-graphene probe. When integrated into AuE, the probe exhibited remarkable sensitivity and consistent detection accuracy, achieving a notably low detection limit of 63.2 fM.

The effectiveness of the sensor extends to the assessment of 5hmC levels in real biological specimens, including various primary tissues from WT or TET knockout mice, mouse models of hepatocellular carcinoma, and human cancer cell lines. Its straightforward synthesis in a cost-effective manner, coupled with its capacity to operate without the need for pretreatment or labeling with affinity molecules or antibodies, renders it a cutting-edge asset for epigenetic research as well as clinical applications for early cancer detection, prognosis evaluation, and tailored personalized therapy. In conclusion, we have presented a graphene-based sensor that demonstrates remarkable selectivity, sensitivity, and reproducibility for quantifying DNA hydroxymethylation levels. The translation of these findings into clinical settings warrants further optimization and validation across a broader spectrum of biological samples.

## CRediT authorship contribution statement

**Habibulla Imran:** Writing – original draft, Methodology, Investigation, Formal analysis, Conceptualization. **Hyun-ji Lee:** Methodology. **Asrar Alam:** Investigation. **Jungeun An:** Writing – review & editing, Supervision, Funding acquisition. **Myunggon Ko:** Writing – review & editing, Supervision, Funding acquisition, Conceptualization. **Sooman Lim:** Writing – review & editing, Supervision, Project administration, Funding acquisition, Conceptualization.

## Declaration of competing interest

The authors declare that they have no known competing financial interests or personal relationships that could have appeared to influence the work reported in this paper.

## Data Availability

Data will be made available on request.
