# Supplementary material for: Ultrasensitive detection of 5-hydroxymethylcytosine in genomic DNA using a graphene-based sensor modified with biotin and gold nanoparticles
Source: Mater Today Bio. 2024 Jun 11;27:101123. doi: 10.1016/j.mtbio.2024.101123 (PMC11234158; doi:10.1016/j.mtbio.2024.101123)
Supplement: Multimedia component 1 [file mmc1.docx]

**Supporting Information**

**Ultrasensitive detection of 5-hydroxymethylcytosine in genomic DNA using a graphene-based sensor modified with biotin and gold nanoparticles**

Habibulla Imran^a,1^_,_ Hyun-ji Lee^b,1^, Asrar Alam^c,d^, Jungeun An^e,^*, Myunggon Ko^b,^*, and Sooman Lim^a,^*

^a^Department of Flexible and Printable Electronics, LANL-JBNU Engineering Institute, Jeonbuk National University, Jeonju, 54896, Republic of Korea

^b^Department of Biological Sciences, Ulsan National Institute of Science and Technology, Ulsan 44919, Republic of Korea

^c^Mycronic AB, Nytorpsvägen 9, Täby, 183 53 Sweden

^d^Wallenberg Initiative Materials Science for Sustainability (WISE), Department of Fibre and Polymer Technology, School of Engineering Sciences in Chemistry, KTH Royal Institute of Technology, Teknikringen 56, Stockholm, 10044 Sweden

^e^Department of Life Sciences, Jeonbuk National University, 567 Baekje-daero, Jeonju 54896, Republic of Korea

^1^These authors contributed equally to this work.

*Co-corresponding authors

J. An

E-mail: jan@jbnu.ac.kr

Dr. Myunggon Ko

Email: mgko@unist.ac.kr

Dr. Sooman Lim

Email: smlim@jbnu.ac.kr

**Experimental**

*2.1. Materials*

The pencil rod was purchased from a local supplier (sharp HB pencil leads with 0.7 mm diameter and 66 mm length). Potassium ferricyanide (ACS reagent, ≥ 99.0%), potassium ferrocyanide (ACS reagent, 98.5–102.0%), potassium chloride (for molecular biology, ≥ 99.0%), Bt (≥ 99% (HPLC), lyophilized powder), gold (III) chloride trihydrate (≥ 99.9%, trace metals basis), electrode polishing kit (PK-4), ethanol (200 proof, ACS reagent, ≥ 99.5%), sulfuric acid (ACS reagent, 95.0–98.0%), hydrogen peroxide (35%), and dimethylformamide (suitable for HPLC, ≥ 99.9%) were purchased from Sigma–Aldrich, USA. These reagents, all conforing to analytical grade, were used as provided without additional purification. Deionized (DI) water was used across all aqueous solutions and cleansing procedures. All solutions were prepared using ultrapure water (18 MΩ cm), produced by a micro pure HIQ water purifying system. Adjustable direct current (DC) power supplies (0–30 V) were purchased from Toyotech, Republic of Korea.

*2.2.* *One-pot direct electrochemical exfoliation and biotin-AuNPs functionalization of graphene using a pencil graphite rod*

Simultaneous electrochemical exfoliation and functionalization of graphene with biotin (Bt) and gold nanoparticles (AuNPs) were performed by applying a steady DC potential between two pencil graphite rods submerged in 5 mL of deionized water (DI water) containing 1 mM Biotin and gold chloride (**Scheme 1**). The rods, positioned parallel at a 1 cm distance, were subjected to various DC potentials—10, 15, 20, and 25 V—over different durations to optimize the exfoliation and functionalization processes. The times were adjusted based on voltage to 68, 57, 49, and 16 hr respectively (**Figure S1**). Variables such as voltage and duration were found to affect the exfoliation rate, conductivity, and morphology of the graphene sheets. Upon completion of exfoliation, a black precipitate formed, which was sonicated for 1 min to achieve a uniform dispersion. This dispersion underwent triple centrifugation at 10,000 rpm for 20 min in DI water and a final spin in ethanol. The product was then dried at 50 °C overnight, yielding the functionalized graphene material.

*2.3.* *Sensor device fabrication*

A sensor device was constructed using electrochemically exfoliated and functionalized graphene (Bt-AuNP-G). We dispersed 1.5 mg of Bt-AuNP-G in 1 mL of dimethylformamide (DMF) and sonicated it to achieve a uniform solution. Then, 3 µL of this dispersion was drop-casted onto the surface of a cleaned gold electrode (AuE) and left to dry at 50 °C for 2 hr. The cleaning process for the AuE involved a 30-min piranha solution treatment, followed by a thorough rinse with DI water [1]. After preparing the electrode surface, 5 µL of 5hmC-containing DNA was applied to the electrodes and incubated for 1 hr at 35 °C in a hot air oven. Post-incubation, any unbound 5hmC-DNA was washed off using a blank KCl solution. To evaluate the sensor's selectivity, DNA samples containing either C, 5mC, or 5hmC were applied to similarly prepared electrodes and treated under identical conditions. After incubation, the surfaces were cleansed with blank buffer solution to eliminate unbound DNA. Cyclic voltammetry (CV) was performed on the prepared electrodes in a solution containing a redox probe—10 mM K_3_[Fe(CN)_6_]/K_4_[Fe(CN)_6_] in 100 mM KCl—at a scan rate of 50 mV/s to assess the electrochemical response. As controls, Au electrodes modified with Bt-G-5hmC-DNA and AuNP-G-5hmC-DNA were also fabricated using the same protocol.

*2.4.* *Characterization of the sensor materials*

The synthesized sensor materials were extensively characterized to determine their chemical structure and morphology. X-ray diffraction (XRD) patterns were obtained using a Rigaku diffractometer and surface topography was examined with field emission scanning electron microscopy (FE-SEM, Zeiss SUPRA 40VP). Raman spectra, covering the range from 300 to 3000 cm^−1^, were recorded using Nanophoton Raman spectroscopy (Raman Touch). For more detailed structural analyses, we employed transmission electron microscopy (TEM, Hitachi H–7650, Japan) and X-ray photoelectron spectroscopy (XPS, Nexsa XPS System, Thermo Fisher Scientific, UK). These characterization were performed at the facilities available at the Center for University-Wide Range Research Facilities (CUWRF), located at Jeonbuk National University in the Republic of Korea.

*2.5.* *Electrochemical measurements and quantification of 5hmC levels*

CV and linear sweep voltammetry (LSV) assays were conducted on a SP-150 model Bio-Logic workstation (France) equipped with EC-lab 11.25 software. The electrochemical setup utilized a standard three-electrode configuration: a gold electrode with a 3 mm diameter and 65 mm length, coated with the synthesized material, served as the working electrode; a platinum wire with a 0.5 mm diameter and 5 cm length functioned as the counter electrode; and an Ag/AgCl electrode was employed as the reference. The electrochemical behavior was assessed in a solution containing either a redox probe of 10 mM K_3_[Fe(CN)_6_]/K_4_[Fe(CN)_6_] in 100 mM KCl, with measurements conducted at a scan rate of 50 mV/s. The K_3_[Fe(CN)_6_]/K_4_[Fe(CN)_6_] served as an electrolyte to evaluate the adsorption conformation of 5hmC-DNA on the modified gold electrode, while KCl was used to verify the oxidation of the 5hmC-DNA modified electrode. Quantification of 5hmC was performed using standard oligonucleotides with known 5hmC concentrations, generating a standard curve from peak current values against 5hmC amounts (Figure 3c). Using the linear regression equation (y = 0.0008x + 0.0039, *R²* = 0.9993) derived from the standard curve, we calculated the 5hmC levels in genomic DNA samples by substituting the obtained peak current values from electrochemical measurements into the regression equation.

*2.6.* *Synthesis of oligonucleotides containing distinct cytosine variants*

Double-stranded oligonucleotides containing C, 5mC, and 5hmC were synthesized using a polymerase chain reaction (PCR) as described elsewhere [2,3]. For the PCR amplification, we used 0.2 mM of each standard nucleoside triphosphate (dATP, dTTP, dGTP) alongside the corresponding cytosine derivative—dCTP, 5-mdCTP, or 5-hmdCTP (Bioline). Post-amplification, the PCR products that contained 5hmC underwent a sodium bisulfite treatment to convert 5hmC into cytosine-3-methylenesulfonate (CMS), using an EpiTect bisulfite kit (QIAGEN) according to the manufacturer’s instructions. The successful incorporation of the different cytosine analogs into the PCR products was validated through agarose gel electrophoresis and dot blot analyses with specific antibodies against 5mC, 5hmC, or CMS.

*2.7.* *Conditional deletion of Tet genes in mouse hepatocytes*

All experiments were performed using C57BL/6 mice. Mice with *LoxP*-flanked *Tet1*, *Tet2*, and *Tet3* genes (designated *Tet1^fl/fl^, Tet2^fl/fl^, and Tet3^fl/f^*) [4-6] were obtained courtesy of Dr. Anjana Rao from the La Jolla Institute, CA, USA. Albumin-Cre transgenic mice, expressing Cre recombinase exclusively in hepatocytes [7], were provided by Dr. Hyug Moo Kwon from the Ulsan National Institute of Science and Technology (UNIST), Ulsan, Korea, originally sourced from the Jackson Laboratory. To induce the targeted deletion of the three *Tet* genes in hepatocytes, we crossed the triple-floxed mice (*Tet1/2/3^fl/fl^*) with Albumin-Cre mice, resulting in offspring carrying *Tet1/2/3^fl/fl^ Albumin-Cre* alleles. The mice were housed in a regulated environment with a consistent temperature of 22 ± 2 °C and a 12-hour light/dark cycle. The breeding and care of the animals were conducted in the In Vivo Research Center (IVRC) at UNIST, adhering to specific pathogen-free standards. All experimental procedures were approved by the Institutional Animal Care and Use Committee of UNIST (approval numbers UNISTACUC-17-35 and 20–23). Moreover, all experiments involving live mice conformed to the institution's ratified guidelines.

*2.8.* *Generation of mouse models with primary hepatocellular carcinoma*

Hepatocellular carcinoma (HCC) was induced in mice following the established procedures [8].

Specifically, male mice at the age of 2 weeks were subjected to a single intraperitoneal dose of diethylnitrosamine (DEN) (25 mg/kg, obtained from Sigma, product #N0756), a potent carcinogenic agent known to reliably induce HCC in a living organism. After nine months, the mice were humanely euthanized, and samples of both tumor (T) nodules and adjacent non-tumor (NT) liver tissues were excised for analysis. This enabled direct comparative studies between matched T and NT samples obtained from the same individual mice. The mice were housed in a temperature-controlled (22 ± 2 °C) facility under a 12-h light/dark cycle.

*2.9.* *Extraction of genomic DNAs and quantification of 5hmC levels*

Isolation of genomic DNAs, bisulfite treatment, and quantification of 5hmC by dot blot analysis were performed as previously described [2,9]. Briefly, cells were lysed and digested with 200 μg/mL proteinase K at 55 °C overnight. Tissues isolated from murine models were lysed using TissueLyser LT in lysis buffer (Qiagen) before proceeding to proteinase K digestion. Genomic DNA was then purified using the conventional phenol-chloroform extraction technique. Prior to downstream applications, extracted DNA was mechanically fragmented by vigorous pipetting 20–30 times, as documented in previous studies [2,5,9,10]. For dot blot analysis, two-fold serial dilutions of denatured DNA samples were blotted onto a nitrocellulose membrane using a Bio-Dot apparatus (Bio-Rad) according to the manufacturer’s instructions, followed by incubation with anti-CMS (provided by Dr. Anjana Rao, La Jolla Institute, USA) overnight at 4 °C. Toluidine blue staining with 0.02% toluidine blue in 0.3 M sodium acetate (pH 5.2) confirmed equal DNA deposition on the membrane. Quantification of 5hmC was conducted using standard oligonucleotides with a known CMS concentration (6.156 pmol of CMS per 10 ng of DNA), as described previously [2,9], creating a standard curve from signal intensities against CMS amounts. Then, using the linear portion of the standard curve, we measured the amount of CMS (and thus 5hmC) in the genomic DNA samples [9].

*2.10.* *Cell culture*

The C4-2B prostate cancer cell line and RWPE-1 normal prostate epithelial-like cell were obtained from the American Type Culture Collection (ATCC). C4-2B cells were cultured in RPMI 1640 supplemented with 10% fetal bovine serum (Gibco). RWPE-1 was cultured in keratinocyte serum-free medium (K-SFM) supplemented with BPE and EGF (Gibco) according to the manufacturer’s instructions. All growth media were supplemented with 1X penicillin/streptomycin (Gibco). Cells were maintained at 37 °C in a 5% CO_2_ atmosphere and tested negative for mycoplasma contamination.

*2.11. Statistical analyses*

All data were presented as the mean ± SEM. Statistical significance was assessed using a two-tailed, unpaired Student’s t-test using GraphPad Prism 8.0 software (GraphPad, CA, USA). **P <* 0.05, ***P <* 0.01, ****P <* 0.001, and *****P <* 0.0001 were considered significant.

**
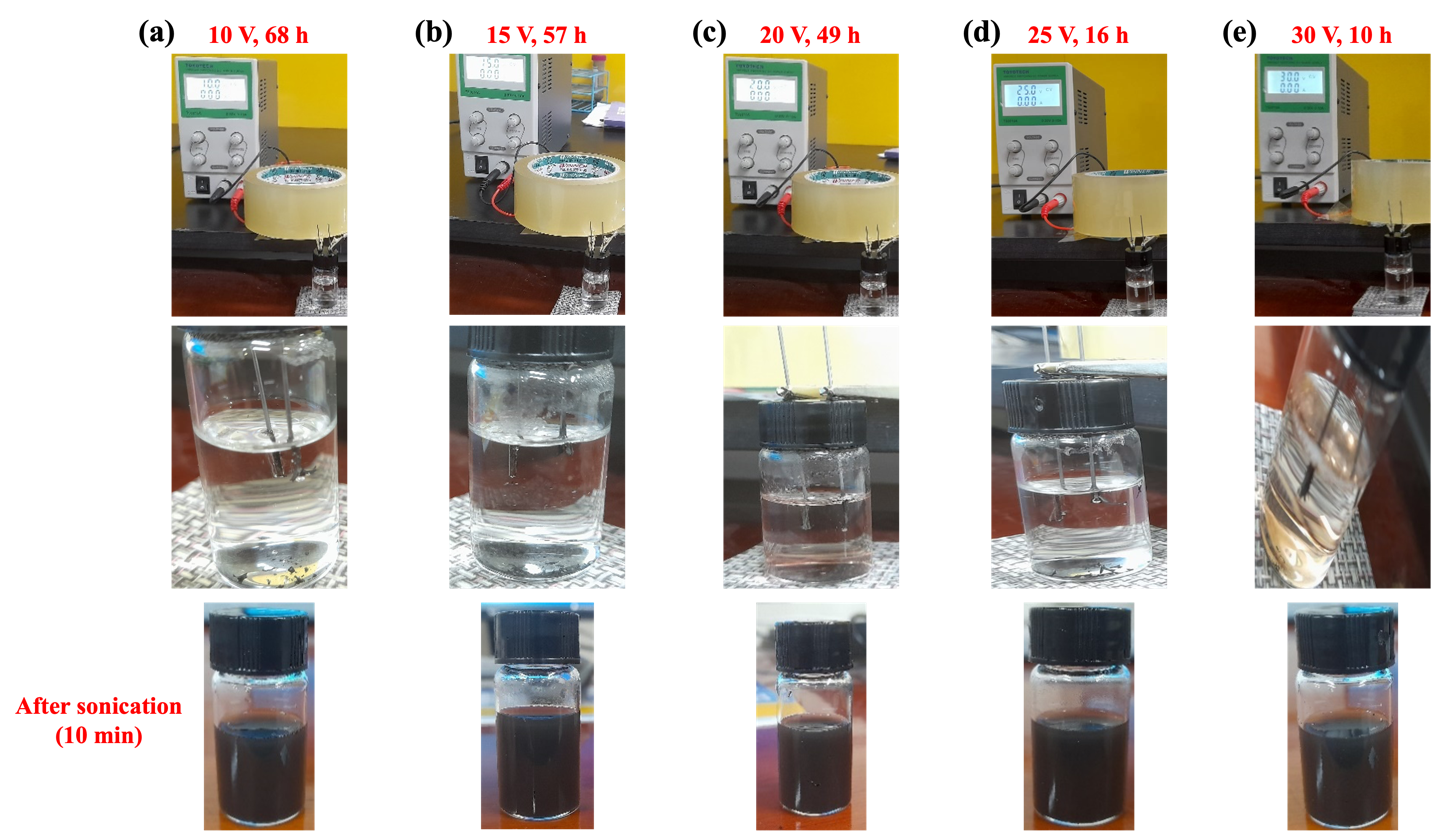
**

**Figure S1**. Synthesis of electrochemically exfoliated and functionalized graphene from a pencil rod under various constant DC potentials in an aqueous medium.


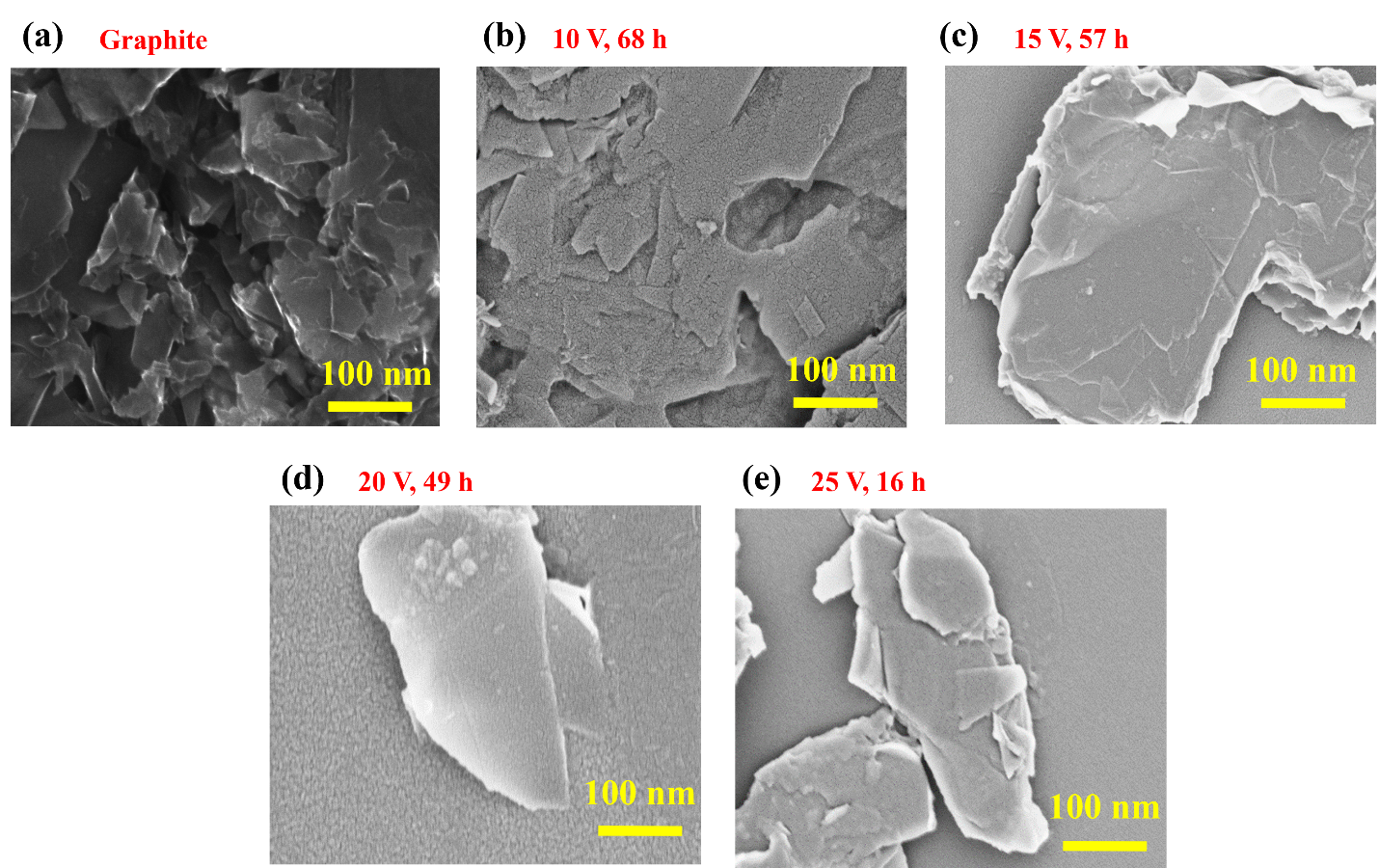


**Figure S2.** SEM images of (a) pencil graphite and (b-e) graphene sheets electrically exfoliated under varying voltages as shown in Figure S1.


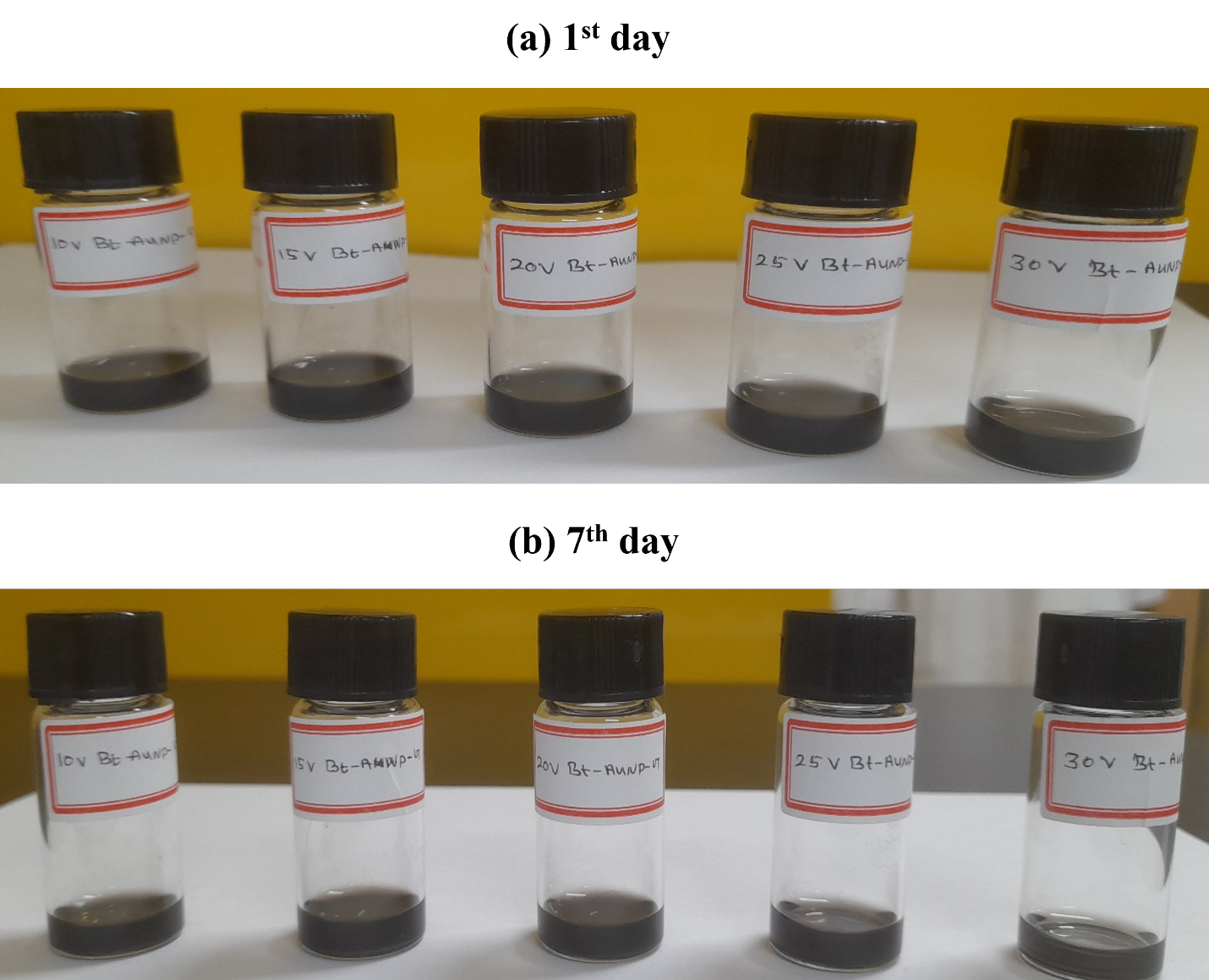


**Figure S3.** Stability of the sonicated, exfoliated graphene powders suspended in dimethylformamide (DMF). (a) The graphene dispersions immediately after preparation. (b) The same dispersions after seven days**.**


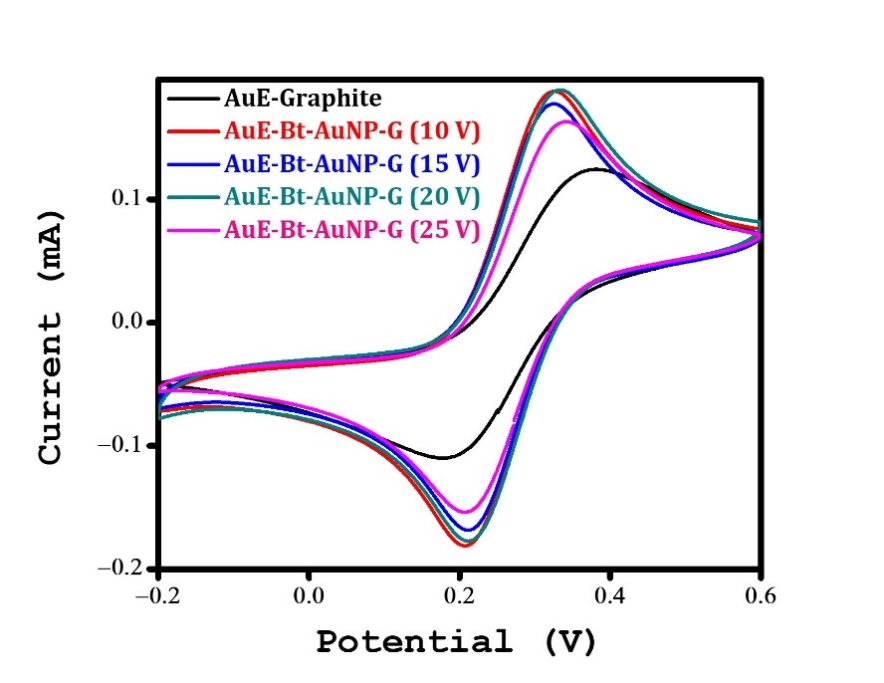


**Figure S4.** CV behavior of the AuE modified with pencil graphite (*black*) or electrochemically exfoliated and Bt-AuNP functionalized graphene prepared at 10 (*red*), 15 (*blue*), 20 (*dark cyan*) and 25 (*magenta*) V, respectively. Measurements were conducted in an electrolyte solution consisting of 10 mM K_3_[Fe(CN)_6_]/K_4_[Fe(CN)_6_] in 100 mM KCl, using a scan rate of 50 mVs^-1^.

**Table S1.** Voltammetric data from experiments involving Au electrodes modified with pencil graphite or electrically exfoliated and Bt-AuNP functionalized graphene, which relate to Figure S4. CV measurements were conducted in a solution of 10 mM K_3_[Fe(CN)_6_]/K_4_[Fe(CN)_6_] at a scan rate of 50 mVs^-1^.


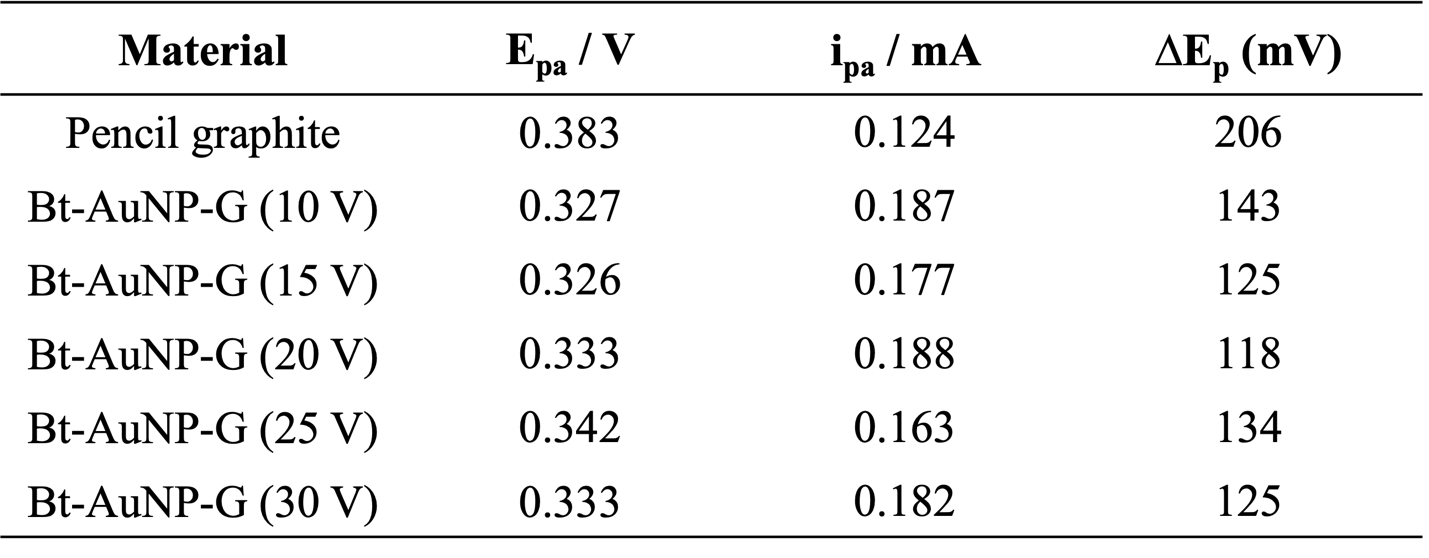


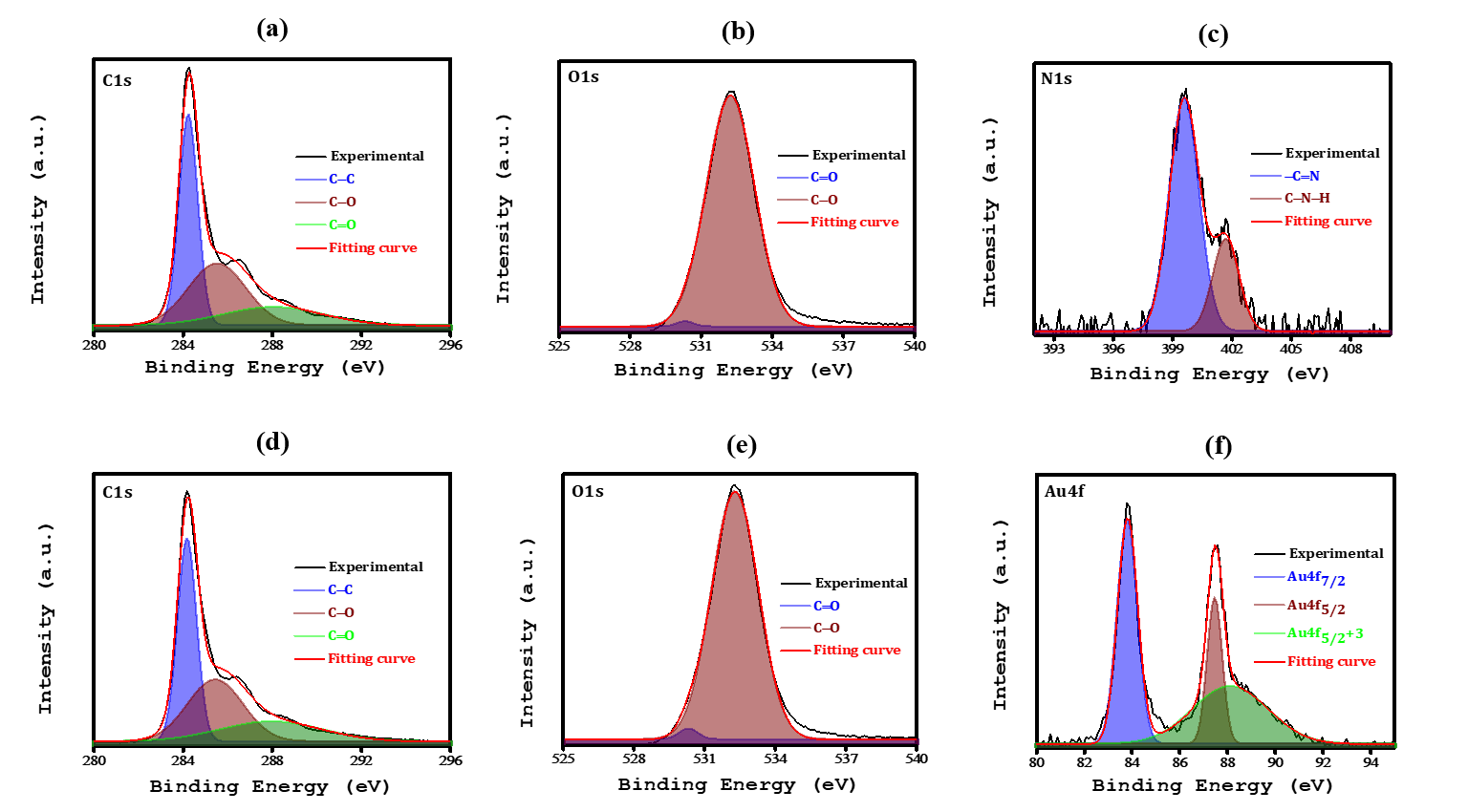


**Figure S5.** XPS spectrum of electrochemically exfoliated graphene functionalized with biotin (Bt-G, a-c) or gold nanoparticles (AuNP-G, d–f).


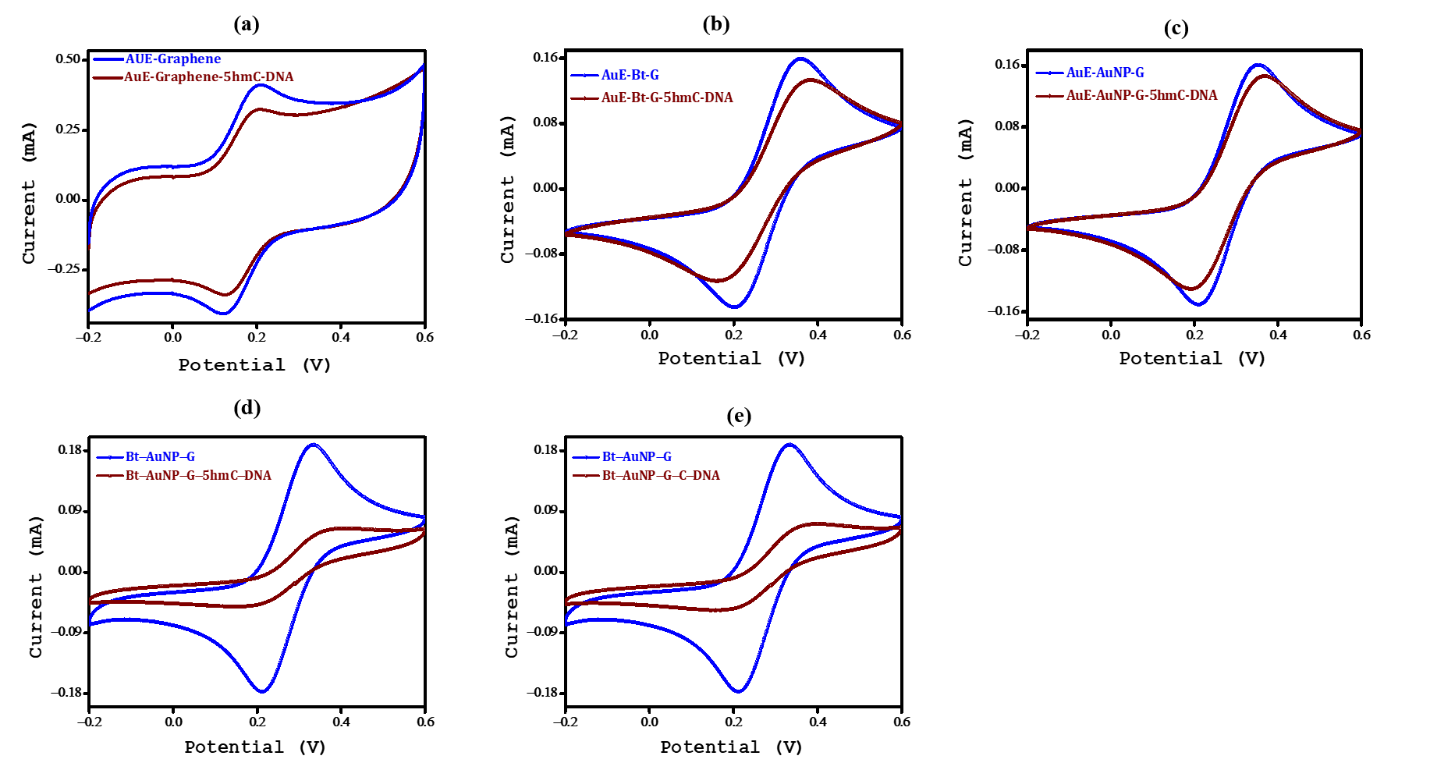


**Figure S6.** CV behavior of gold electrode modified with a) graphene, b) Bt-G, c) AuNP-G, and d) Bt-AuNP-G before or after immobilization with 5hmC-containing DNA (5hmC-DNA). e) Bt-AuNP-G modified Au electrode before or after immobilization with cytosine-containing DNA (C-DNA). All measurements are made in the presence of 10 mM K_3_[Fe(CN)_6_]/K_4_[Fe(CN)_6_] in 100 mM KCl at a scan rate of 50 mVs^-1^.


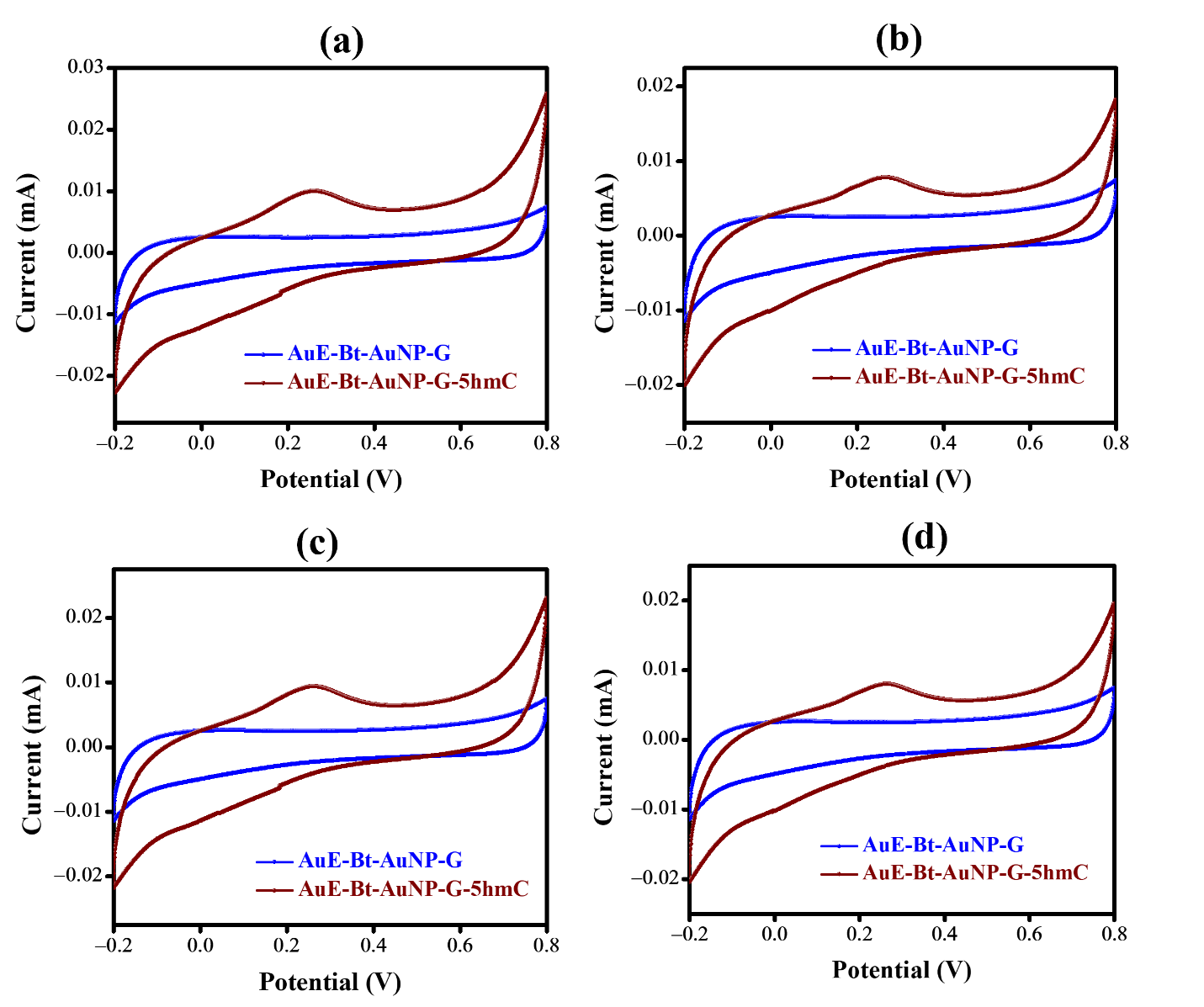


**Figure S7.** Reproducibility study of CV measurements conducted with independently prepared Bt-AuNP-G-modified Au electrodes with or without immobilization of 5hmC-DNA. Measurements were carried out in a 100 mM KCl solution at a scan rate of 50 mVs^-1^.


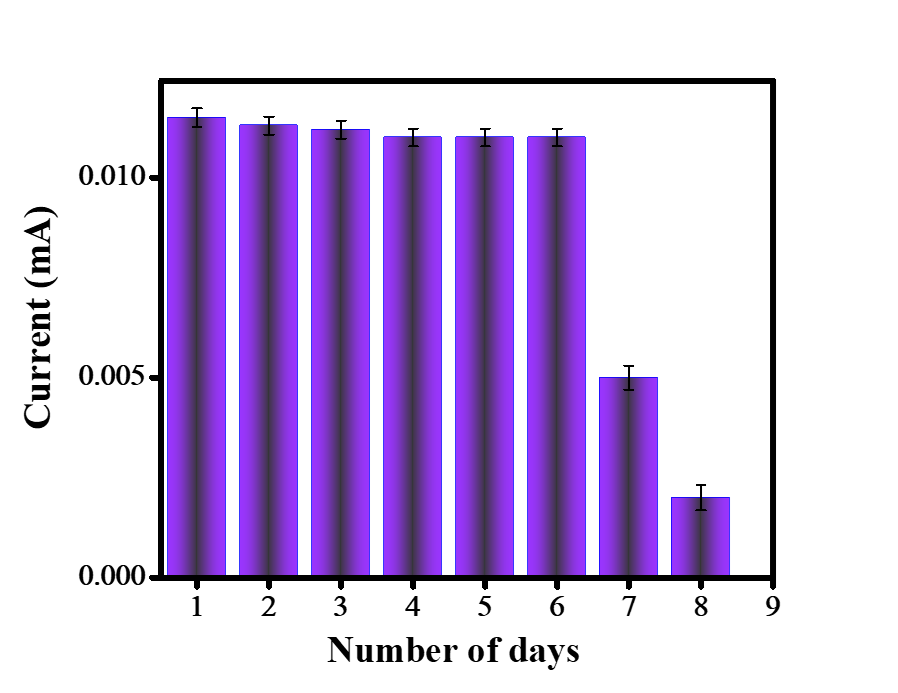


**Figure S8**. Stability assessment of an AuE-Bt-AuNP-G-5hmC-DNA-modified Au electrode through CV analysis. Measurements were conducted in a 100 mM KCl at a scan rate of 50 mVs^-1^. The graph presents the anodic peak current plotted over several days to evaluate the electrode's long-term electrochemical stability. The error bars were derived from three independent measurements. Data: Mean ± standard deviation (S.D.).

**Table S2.** Comparison of the performance of the developed sensor with other 5hmC detection sensors

|  | **Detection methods** | **Linear detection range** | **LOD** | **Reference** |
| --- | --- | --- | --- | --- |
| Electrochemi-  luminescence | 5hmC antibody and PDDA-CNTs nanocomposite | 0.01–2 nM | 2.3 pM | [11] |
|  | Covalent bonding of CH_2_OH and thiol-functionalized Fe_3_O_4_ magnetic beads | 0.01–500 nM | 2.86 pM | [12] |
|  | Fe_3_O_4_@SiO_2_ nanoparticles and PAMAM dendrimers | 0.1–30 nM | 0.047 nM | [13] |
|  | Gold nanoclusters-H_2_O_2_ system | 0.01–50 nM | 3.46 pM | [14] |
|  | PAMAM-nanosilver-nitrogen doped graphene nanocomposite | 0.01–30 nM | 2.47 pM | [15] |
|  | Selective electrogenerated chemiluminescence (ECL) labeling with the specific oxidation of 5-hmC to 5-fC by KRuO_4_ | 0.5–100 pM | 0.14 pM | [16] |
|  | T4 β-glucosyltransferase (β-GT)-mediated 5hmC glycosylation and signal quenching from ferroceneboronic acid | 0.05–10 nM | 16.3 pM | [17] |
| Photoelectro-  chemistry | M. HhaI methyltransferase catalytic covalent bonding | 0.01–100 nM | 4.12 pM | [18] |
|  | In-situ electron donor producing strategy | 0.5–100 nM | 0.167 nM | [19] |
|  | In-situ synthesized Bi_4_NbO_8_Cl@Bi_2_S_3_ heterojunction | 0.3–300 nM | 0.0779 nM | [20] |
|  | Photocurrent inhibition effect of ZnO on MoS_2_/C_3_N_4_ heterojunction | 0.01–200 nM | 2.6 pM | [21] |
| Single-molecule imaging | DNA hybridization-based single-molecule immunofluorescent imaging | - | 100 × 10^−12^ m | [22] |
|  | Chemical labeling-based single-molecule imaging | - | 1 pM | [23] |
|  | Molecular imaging of 5hmC in cell-free DNA | 0.0005-50 ng | - | [24] |
| Fluorescence | Boric acid-functionalized nano-microsphere fluorescent probes | 0–100 nM | 0.167 nM | [25] |
| Capillary electrophoresis | Capillary electrophoresis with laser induced fluorescence | 0.09–90 nM | 0.09 nM | [26] |
| Electrochemistry | T4 β-glucosyltransferase (β-GT)-mediated 5hmC glycosylation and HpaII restriction endonuclease system | 0.5–90 nM | 0.14 nM | [27] |
|  | Anti-5-hmC antibody, biotin functionalized phos-tag, and avidin functionalized alkaline phosphatase | 0.1–30 nM | 32 pM | [28] |
|  | A nozzle-jet printed AuNPs@Ti_3_C_2_ MXene | 0.632–63.2 pM | 0.632 pM | [29] |
|  | A g-C_3_N_4_-modified gold electrode | 0.316–63.2 pM | 0.316 pM | [30] |
|  | A Bt-AuNP-G modified gold electrode | 0.00316–1.58 pM | 63.2 fM | This work |

~~
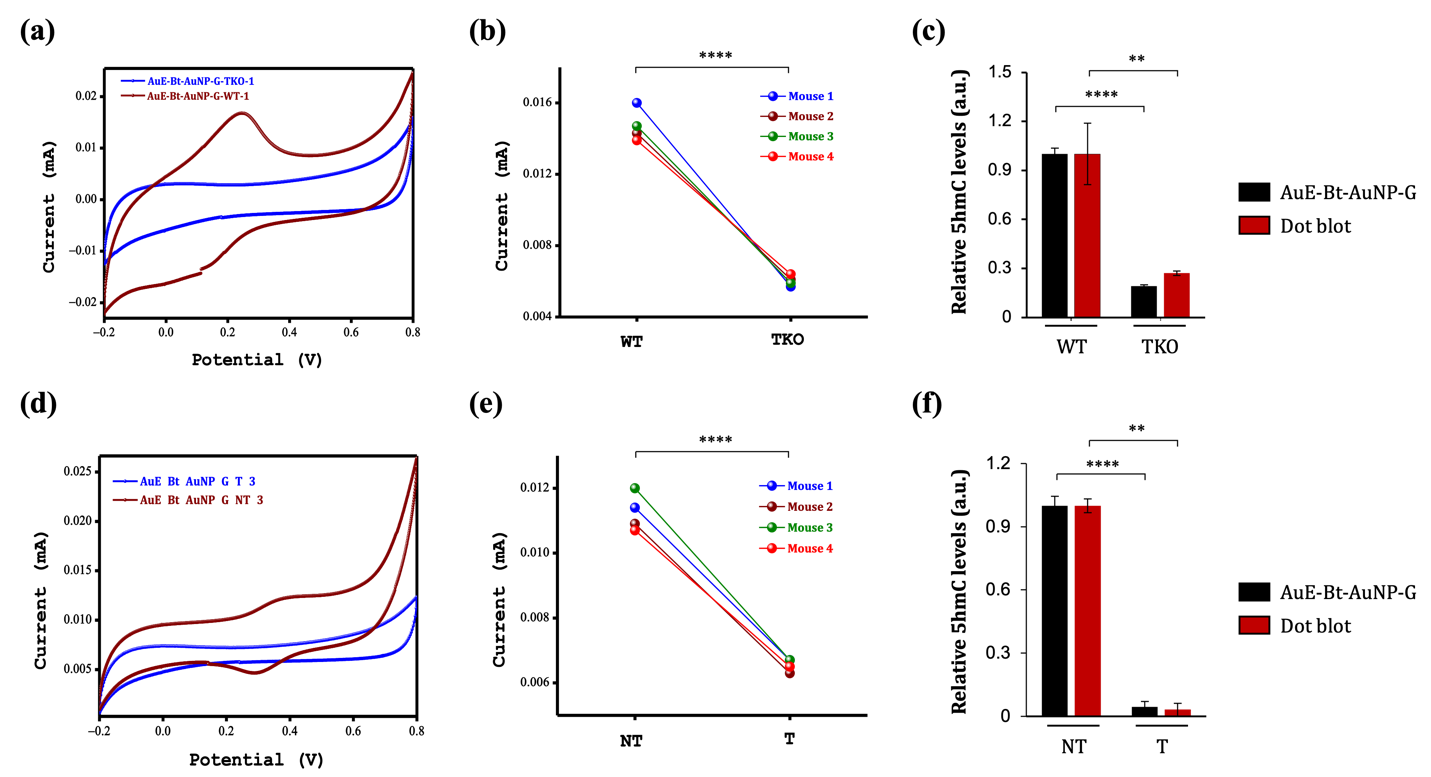
~~

**Figure S9**. a) CV analysis using AuE-Bt-AuNP-G sensor with liver DNA from WT and TKO mice. A representative result from four independent samples. b) A summary of the data in (a), with each dot representing the average of three separate experiments. c) Correlation of results obtained from CV analysis with AuE-Bt-AuNP-G sensor and to those from CMS dot blot. d) CV analysis using AuE-Bt-AuNP-G sensor on DNA samples from tumor (T) and adjacent non-tumor (NT). A representative result from four independent samples. e) A summary of the results in (d), with each dot indicating the mean of three separate experiments. f) Validation of CV results obtained with AuE-Bt-AuNP-G sensor against those from dot blot assays. All CV measurements were performed in 100 mM KCl solution at a scan rate of 50 mVs-1. Data: Mean ± SEM; ** p ≤ 0.01 and **** p ≤ 0.0001 (unpaired Student’s t-test).

**References**

[1] H. Imran, P.N. Manikandan, V. Dharuman, Ultra-sensitive and selective label free electrochemical DNA detection at layer-by-layer self-assembled graphene oxide and vesicle liposome nano-architecture, J. Electroanal. Chem. 835 (2019) 10-21.

[2] Y. Huang, W.A. Pastor, Y. Shen, M. Tahiliani, D.R. Liu, A. Rao, The behaviour of 5-hydroxymethylcytosine in bisulfite sequencing, PLoS One 5(1) e8888.

[3] S.Y. Lee, X. Qi, M. Ko, C.H. Park, J. An, S. Lim, Direct and rapid detection of 5-hydroxymethylcytosine, a novel cancer hallmark in DNA, using electrochemical reaction, Mater. Today Commun. 25 (2020) 101399.

[4] M. Ko, J. An, W.A. Pastor, S.B. Koralov, K. Rajewsky, A. Rao, TET proteins and 5-methylcytosine oxidation in hematological cancers, Immunol. Rev. 263(1) (2015) 6-21.

[5] M. Ko, H.S. Bandukwala, J. An, E.D. Lamperti, E.C. Thompson, R. Hastie, A. Tsangaratou, K. Rajewsky, S.B. Koralov, A. Rao, Ten-Eleven-Translocation 2 (TET2) negatively regulates homeostasis and differentiation of hematopoietic stem cells in mice, Proc. Natl. Acad. Sci. U. S. A. 108(35) (2011) 14566-14571.

[6] J. Kang, M. Lienhard, W.A. Pastor, A. Chawla, M. Novotny, A. Tsagaratou, R.S. Lasken, E.C. Thompson, M.A. Surani, S.B. Koralov, S. Kalantry, L. Chavez, A. Rao, Simultaneous deletion of the methylcytosine oxidases Tet1 and Tet3 increases transcriptome variability in early embryogenesis, Proc. Natl. Acad. Sci. U. S. A. 112(31) (2015) E4236-4245.

[7] J.H. Lee, J.H. Suh, S.Y. Choi, H.J. Kang, H.H. Lee, B.J. Ye, G.R. Lee, S.W. Jung, C.J. Kim, W. Lee- Kwon, J. Park, K. Myung, N.H. Park, H.M. Kwon, Tonicity-responsive enhancer-binding protein promotes hepatocellular carcinogenesis, recurrence and metastasis, Gut 68(2) (2019) 347-358.

[8] R. Tolba, T. Kraus, C. Liedtke, M. Schwarz, R. Weiskirchen, Diethylnitrosamine (DEN)-induced carcinogenic liver injury in mice, Lab. Anim. 49(1 Suppl) (2015) 59-69.

[9] M. Ko, Y. Huang, A.M. Jankowska, U.J. Pape, M. Tahiliani, H.S. Bandukwala, J. An, E.D. Lamperti, K.P. Koh, R. Ganetzky, X.S. Liu, L. Aravind, S. Agarwal, J.P. Maciejewski, A. Rao, Impaired hydroxylation of 5-methylcytosine in myeloid cancers with mutant TET2, Nature 468(7325) (2010) 839-843.

[10] S. Byun, C.H. Lee, H. Jeong, H. Kim, H.M. Kwon, S. Park, K. Myung, J. An, M. Ko, Loss of adipose TET proteins enhances beta-adrenergic responses and protects against obesity by epigenetic regulation of beta3-AR expression, Proc. Natl. Acad. Sci. U. S. A. 119(26) (2022) e2205626119.

[11] Y. Meng, W. Bai, Y. Zhang, H. Sun, Y. Li, Electrogenerated chemiluminescence biosensing method based on 5-hydroxymethylcytosine antibody and PDDA-CNTs nanocomposites for the determination of 5-hydroxymethylcytosine double-stranded DNA, Talanta 210 (2020) 120597.

[12] C. Sui, H. Yin, L. Wang, Y. Zhou, S. Ai, Electrochemiluminescence biosensor for DNA hydroxymethylation detection based on enzyme-catalytic covalent bonding reaction of -CH2OH and thiol functionalized Fe3O4 magnetic beads, Biosens. Bioelectron. 150 (2020) 111908.

[13] W. Jiang, L. Wu, J. Duan, H. Yin, S. Ai, Ultrasensitive electrochemiluminescence immunosensor for 5-hydroxymethylcytosine detection based on Fe3O4@SiO2 nanoparticles and PAMAM dendrimers, Biosens. Bioelectron. 99 (2018) 660-666.

[14] W.J. Jiang, H.S. Yin, Y.L. Zhou, J.L. Duan, H.S. Li, M.H. Wang, G.I.N. Waterhouse, S.Y. Ai, A novel electrochemiluminescence biosensor for the detection of 5-methylcytosine, TET 1 protein and beta-glucosyltransferase activities based on gold nanoclusters-H2O2 system, Sensor. Actuat. B-Chem. 274 (2018) 144-151.

[15] J. Ding, W.J. Jiang, Y.L. Zhou, H.S. Yin, S.Y. Ai, Electrochemiluminescence immunosensor for 5-hydroxymethylcytosine detection based on PAMAM-nanosilver-nitrogen doped graphene nanocomposite, J. Electroanal. Chem. 877 (2020) 114646.

[16] S. Ma, H. Sun, Y. Li, H. Qi, J. Zheng, Discrimination between 5-Hydroxymethylcytosine and 5-Methylcytosine in DNA via Selective Electrogenerated Chemiluminescence (ECL) Labeling, Anal. Chem. 88(20) (2016) 9934-9940.

[17] Y. Zhang, Y. Li, Y. Wei, H. Sun, H. Wang, A sensitive signal-off electrogenerated chemiluminescence biosensing method for the discrimination of DNA hydroxymethylation based on glycosylation modification and signal quenching from ferroceneboronic acid, Talanta 170 (2017) 546-551.

[18] Y.L. Zhou, H.S. Yin, C.J. Sui, Y. Wang, S.Y. Ai, Photoelectrochemical detection of 5-hydroxymethylcytosine in genomic DNA based on M. HhaI methyltransferase catalytic covalent bonding, Chem. Eng. J. 357 (2019) 94-102.

[19] Z.Q. Yang, Y.H. Shi, W.R. Liao, H.S. Yin, S.Y. Ai, A novel signal-on photoelectrochemical biosensor for detection of 5-hydroxymethylcytosine based on in situ electron donor producing strategy and all wavelengths of light irradiation, Sensor. Actuat. B-Chem. 223 (2016) 621-625.

[20] J. Ding, Y. Zhou, Q. Wang, S. Ai, Photoelectrochemical biosensor for DNA hydroxymethylation detection based on the enhanced photoactivity of in-situ synthesized Bi4NbO8Cl@Bi2S3 heterojunction, Biosens. Bioelectron. 194 (2021) 113580.

[21] C. Sui, F. Li, H. Wu, H. Yin, S. Zhang, G.I.N. Waterhouse, J. Wang, L. Zhu, S. Ai, Photoelectrochemical biosensor for 5hmC detection based on the photocurrent inhibition effect of ZnO on MoS2/C3N4 heterojunction, Biosens. Bioelectron. 142 (2019) 111516.

[22] Y. Du, Y. Lai, J-Y. Liu, J. Diao, Epigenetic quantification of DNA 5-hydroxymethylcytosine using DNA hybridization-based single-molecule immunofluorescent imaging, Small Methods, 5 (2021) 2100061.

[23] Y. Wang, X. Hu, J. Long, J. Diao, Epigenetic optical sensing of 5-hydroxymethylcytosine at the single-molecule level, Sensor. Actuat. B-Chem. 358 (2022) 131500.

[24] M. Wu, Y. Xiang, L. Tang, W. Li, X. Wang, Y. Du, W. Zuo, S. Zhang, Q. Hu, M. Wang, J. Diao, J. Wu, Y. Lai, Molecular imaging of aging-related 5-hydroxymethylcytosine in cell-free DNA at the single-copy level, Sensor. Actuat. B-Chem. 402 (2024) 135098.

[25] H.Y. Chen, J.R. Wei, J.X. Pan, W. Zhang, F.Q. Dang, Z.Q. Zhang, J. Zhang, Spectroscopic quantification of 5-hydroxymethylcytosine in genomic DNA using boric acid-functionalized nano-microsphere fluorescent probes, Biosens. Bioelectron. 91 (2017) 328-333.

[26] A.M. Krais, Y.J. Park, C. Plass, H.H. Schmeiser, Determination of genomic 5-hydroxymethyl-2'-deoxycytidine in human DNA by capillary electrophoresis with laser induced fluorescence, Epigenetics 6(5) (2011) 560-565.

[27] H.S. Yin, Z.Q. Yang, H.Y. Wang, Y.L. Zhou, S.Y. Ai, Electrochemical biosensor for hydroxymethylated DNA detection and beta-glucosyltransferase activity assay based on enzymatic catalysis triggering signal amplification, Sensor. Actuat. B-Chem. 243 (2017) 602-608.

[28] Z. Yang, W. Jiang, F. Liu, Y. Zhou, H. Yin, S. Ai, A novel electrochemical immunosensor for the quantitative detection of 5-hydroxymethylcytosine in genomic DNA of breast cancer tissue, Chem. Commun. (Camb) 51(78) (2015) 14671-14673.

[29] K.S. Bhat, S. Byun, A. Alam, M. Ko, J. An, S. Lim, A fast and label-free detection of hydroxymethylated DNA using a nozzle-jet printed AuNPs@Ti3C2 MXene-based electrochemical sensor, Talanta 244 (2022) 123421.

[30] H. Imran, J. An, K. Jang, A. Alam, V. Dharuman, M. Ko, S. Lim, Highly selective and real-time detection of 5-hydroxymethylcytosine in genomic DNA using a carbon nitride-modified gold transducer-based electrochemical sensor, J. Alloy. Compd. 948 (2023) 169715.
